# Sustainable dye removal using fish scale-derived biosorbent: performance and mechanisms

**DOI:** 10.3389/fchem.2025.1711822

**Published:** 2026-03-03

**Authors:** Fatima Ezzahra Atmani, Zouhair Jemali, Safaa Adim, Meriem Kasbaji, Morad Kaddouri, Nadia Anter, Mohammed Al-zharani, Fahd A. Nasr, Ashraf Ahmed Qurtam, Reda El kacmi, Abdelali Bouli, Aziz Hasib

**Affiliations:** 1 Laboratory of Environmental, Ecological and Agro-Industrial Engineering, Faculty of Science and Technologies, Sultan Moulay Slimane University, Beni Mellal, Morocco; 2 Laboratory of Molecular Chemistry, Materials and Catalysis, Faculty of Science and Technologies, Sultan Moulay Slimane University, Beni Mellal, Morocco; 3 Materials Science, Energy and Nanoengineering (MSN) Department, Mohammed VI Polytechnic University, Ben Guerir, Morocco; 4 Laboratory of the Engineering and Applied Technologies, Higher School of Technology, Sultan Moulay Slimane University, Beni Mellal, Morocco; 5 Biology Department, College of Science, Imam Mohammad Ibn Saud Islamic University (IMSIU), Riyadh, Saudi Arabia

**Keywords:** adsorption, bio-based adsorbent, congo red, methylene blue, renewable biomaterials, *Sardina pilchardus* scales

## Abstract

**Introduction:** In the context of sustainable methodologies for wastewater remediation, this study focuses on the development of an efficient and low-cost biosorbent derived from *Sardina pilchardus* fish scales (SPFS) for dye removal applications.

**Methods:** The prepared biosorbent was characterized using physicochemical and morphological analyses to evaluate its surface properties and porosity. Batch adsorption experiments were conducted to investigate the removal of Methylene Blue (MB) and Congo Red (CR), including kinetic, isotherm, thermodynamic, and regeneration studies.

**Results:** The material exhibited a porous structure and rich surface functional groups favorable for adsorption. Fast adsorption kinetics were observed, with equilibrium reached within 30 min for both dyes. The adsorption process followed the pseudo-second-order model, indicating chemisorption as the controlling mechanism. Isotherm analysis showed that the Langmuir-Freundlich model provided the best fit, suggesting a heterogeneous surface with combined monolayer and multilayer adsorption. Maximum adsorption capacities of 187.634 mg/g for CR and 129.694 mg/g for MB were achieved, placing SPFS among the most efficient bio-derived adsorbents reported. Thermodynamic parameters confirmed that the adsorption processes were spontaneous, exothermic, and favorable.

**Discussion:** Although a slight decrease in adsorption efficiency was observed at higher temperatures, the biosorbent demonstrated excellent regenerability and maintained high performance over multiple cycles. These findings highlight the strong potential of fish-scale-derived biosorbents as sustainable, efficient, and reusable materials for dye removal in wastewater treatment.

## 1 Introduction

Freshwater poisoning from industrial effluents, especially synthetic dyes, is a growing environmental problem with significant effects (Tella et al., 2025). Because they can result in cancers, inflammatory reactions, and DNA abnormalities, organic dyes pose a major threat to both economic progress and public health (Khanmohammadi et al., 2019). MB and CR are popular organic dyes used on wood, silk, cotton, and paper. The MB and CR are found in the aqueous environment. One typical thiazine (cationic) dye is MB, specifically (C_16_H_18_N_3_CIS). CR and other artificial anionic azo colorants, specifically (C_32_H_22_N_6_Na_2_O_6_S_2_) (Zheng et al., 2021), are commonly used in dyeing industries (Costa et al., 2020). Its frequent discharge into natural water systems is concerning due to its persistent and toxic nature, posing potential risks to aquatic ecosystems and human health (Manzoor et al., 2024). At very low amounts, Methylene Blue (MB) and Congo Red (CR) are hazardous to the environment because they restrict light penetration in aquatic environments, which interferes with photosynthetic activity and lowers the amount of oxygen in the water (Bopape et al., 2024).

As a result, environmental research is now focused on developing efficient and affordable techniques for eliminating MB from wastewater (Su et al., 2024). Traditional methods for dye removal often present limitations, including high operational costs, the generation of toxic by-products, and challenges in scalability (Sahu and Poler, 2024). Therefore, the quest for more sustainable, efficient, and economical methods has led researchers to explore biosorption as a viable alternative (Hussain et al., 2024). Adsorption is becoming more and more well-known as an economical and effective technique for wastewater dye removal (Bilal et al., 2022).

Biosorption utilizes natural and waste materials of biological origin to adsorb contaminants from aqueous solutions (Karnwal, 2024). Researchers have concentrated on affordable alternatives by utilizing locally sourced precursor materials from renewable natural resources. Among the different biosorbents studied, fish scales have demonstrated encouraging promise because of their accessibility, affordability, and the existence of functional groups that can bind dye molecules (Ighalo, 2020). Many fish species are eaten daily across the globe (Plant, 2021). Consequently, much waste is produced at fish shops and in marine and processing facilities, representing 50%–70% of the original materials (Krishna et al., 2013). Fish scales are composed primarily of collagen and hydroxyapatite. They are a naturally porous structure and functional groups that can facilitate the binding of dye molecules, making them a potentially efficient and sustainable biosorbent (Kabir, 2018; Nouj et al., 2022). Additionally, sardines (*Sardina pilchardus*) rank among the most heavily fished species, with an annual catch of 10,000 tons (Benyounes et al., 2017). Most of the waste generated includes heads, skin, bones, and scales. This waste is discarded without recycling, leading to potential environmental issues. However, by utilizing current biotechnology to process the waste from seafood processing industries, it could be transformed into highly valuable products, turning it into a potential bioresource (Arvanitoyannis and Kassaveti, 2008).

The purpose of this study is to evaluate whether *Sardina pilchardus* fish scales (SPFS) can be employed as an effective biosorbent for removing CR and MB from wastewater. The study aims to maximize adsorption by taking into account solution pH, contact time, biosorbent dosage, and initial dye concentration. It also investigates kinetics, thermodynamics, and the influence of physicochemical characteristics on efficacy. Understanding the adsorption behavior of MB and CR onto fish scales is crucial for developing feasible wastewater treatment systems. Kinetic models such as pseudo-first-order, pseudo-second-order, and intraparticle diffusion will be used, and equilibrium will be assessed using isotherm models such as Langmuir, Freundlich, and Temkin. Thermodynamic parameters (enthalpy, entropy, and Gibbs free energy) will provide information about viability and fundamental properties. The projected results may contribute to environmental engineering by providing an eco-friendly, cost-effective solution for dye-contaminated water. This research promotes sustainable growth and the regenerative economy by transforming fisheries trash into a useful resource.

## 2 Materials and methods

### 2.1 Reagents and materials used

Methylene blue (MB, C_16_H_18_CIN_3_S, >82%), Congo Red (CR, C_32_H_22_N_6_Na_2_O_6_S_2_, ≥98%), sodium hydroxide (NaOH, ≥98%), phosphoric acid (H_3_PO_4,_ ≥85%), and hydrochloric acid (HCl, ≥37%) were made available by Sigma Aldrich.

### 2.2 Collection of raw fish scales

The SPFS were obtained at Beni Mellal, Morocco. The scales were cleansed with ethanol and tap water several times, immersed in distilled water for 12 h, and then rinsed with distilled water again to remove any remaining organic and inorganic impurities. After that, they were baked in an oven at 80 °C until they were crispy. After drying, a mechanical grinder was used to grind the scales into a fine powder. After being sieved to a consistent 1 mm particle size, this powder was kept at room temperature in plastic bags. FTIR and TGA tests were then used to examine the dried scales.

### 2.3 Preparation of bio-sorbents

**FIGURE 1:**
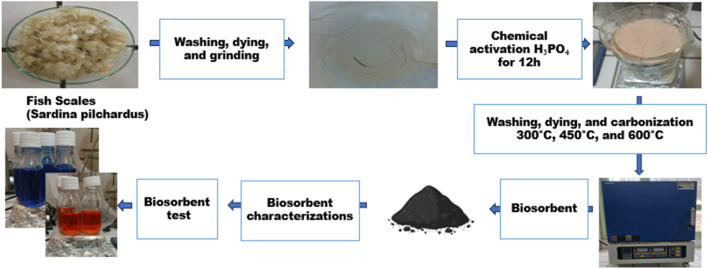
Procedure for the chemical activation process of fish scale–derived biosorbents.

The SPFS is prepared with phosphoric acid (H_3_PO_4_), which promotes surface activation by dehydration and crosslinking reactions with biopolymers such as collagen and chitin. It introduces phosphate and oxygen-containing functional groups, enhances porosity, and prevents excessive shrinkage during carbonization. For a defined duration, the dried fish scales are soaked in the acid solution, which serves as an activating agent to achieve complete impregnation. This procedure improves the material’s porosity and surface functionality, making it more suitable for adsorption. The impregnation ratio (w/w) is mentioned in Table 1 for 12 h. After impregnation, the scales are washed with distilled water to remove acid and the base residuals and dried at 100 °C for 24 h. The scales are then subjected to a controlled heating process, often at temperatures between 300 °C, 450 °C, and 600 °C, in an oven to activate the bio-adsorbent. This thermal activation process helps develop a porous structure within the scales, enhancing their surface area and adsorption capacity. The bio-adsorbent was stored in air-tight containers for further use (Figure 1) (Xie et al., 2023). The samples were examined using FTIR, TGA/DTG, XRD, and SEM.

**TABLE 1:** Parameters for biosorbent preparation: Impregnation ratio, duration, and activation.

Impregnation ratio	Température (°C)	Impregnation time (h)
1–0.5	300	1
1–0.5	450	1
1–0.5	600	1
1–0.5	300	2
1–0.5	450	2
1–0.5	600	2
1–1	300	1
1–1	450	1
1–1	600	1
1–1	300	2
1–1	450	2
1–1	600	2

### 2.4 Preparation of dye solutions

MB and CR are synthetic dyes that are widely used in a range of applications. Including biological staining, textile dyeing, and adsorption investigations in wastewater treatment. Both dyes are often researched for adsorption onto various materials, including biosorbents, activated carbon, chitosan, and metal oxide nanoparticles, to remove them from wastewater. A stock solution is created by dissolving 1 g of MB and CR in 1 L of distilled water. To create a calibration curve for figuring out the remaining MB concentrations during fish scale adsorption tests, this stock solution was then diluted. A V-1200 visible spectrophotometer (VWR) was used to detect the absorbance.

### 2.5 Batch equilibrium studies

The produced biosorbent’s ability to remove CR and MB from aqueous solutions through batch adsorption was assessed. Both MB and CR utilized an initial dye dose of 20 mg/L for these studies. The adsorption of MB and CR was assessed by combining various concentrations of the dye solution with 5–60 mg of fish scales from Sardina pilchards in 20 mL Erlenmeyer flasks. MB and CR adsorption was examined with several experimental parameters, such as contact time (from 2 to 120 min), initial dye concentrations (from 5 to 100 mg/L). pH values (between 2 and 12), and temperatures (from 15 °C to 50 °C). Using 0.1 M HCl or 0.1 M NaOH, the pH of the MB and CR solutions was carefully brought to the levels needed for the adsorption tests. A magnetic stirrer was used to fully mix the solutions once the pH was adjusted to guarantee uniformity throughout the combination. A small amount of the aqueous solution was taken at various points in time. A UV-visible spectrophotometer and an external calibration curve were used to measure the amount of MB and CR that remained in the solution following adsorption. Absorbance readings were taken at a peak wavelength of 664 nm for MB and 500 nm for CR. Equations 1–3 were used to compute the amount of dye retained per gram of adsorbent at equilibrium (Qe), the adsorption capacity at any given time (t) and the bioadsorbent’s removal efficiency (R%).

$$\text{Qe}=\frac{C_{i}-C_{f}}{m}\times V$$ (1)

$$Q_{t}=\frac{C_{i}-C_{t}}{m}\times V$$ (2)

$$R\%=\frac{C_{i}-C_{f}}{C_{i}}\times V$$ (3)

The initial concentrations of MB and CR are denoted by C_i_ and C_f_ (mg/L), respectively, in this framework. The dye concentration at a specific time t is denoted by C_t_ (mg/L). Furthermore, m (g) denotes the mass of the biosorbent that was utilized, whilst V (L) represents the volume of the dye solution.

### 2.6 Characterizations of bio-sorbents

The elemental composition of bio-sorbent derived from fish scales was determined using a CHNS analyzer (model 932, LECO, Michigan, United States of America). The analysis focused on elements such as carbon (C), nitrogen (N), calcium (Ca), phosphorus (P), oxygen (O), and sulfur (S) (Fadeeva et al., 2008).

FT-IR analysis was performed to determine the structures and functional groups present in the biosorbent. The fibers’ FTIR spectra were recorded using an FTIR spectrometer (JASCO-4600, CLASS 1 LASER PRODUCT) within the infrared range of 400–4,000 cm^−1^, with a resolution of 4 cm^−1^. Based on literature references, characteristic bands corresponding to the materials studied were identified (Manoj Kumar Reddy et al., 2013).

To evaluate the materials’ morphology, a scanning electron microscopy (SEM) analysis was conducted using a Jeol JSM-7000 F BRUKER at 15 kV. Additionally, the fundamental composition of the bio-sorbent was analyzed with a CHNS analyzer (model 932, LECO, Michigan, United States of America) (Relucenti et al., 2021).

The crystallinity of the materials was assessed using a BRUKER X-ray diffractometer (D8 ADVANCE). The measurements were taken over two ranges, 5°–50°, with a scan rate of 1°/min (Bonilla-Jaimes et al., 2016). The crystallinity index (CrI) values were calculated with Equation 4.

$$\text{CrI}\left(\%\right)=\frac{I_{crystalline}-I_{amorphous}}{I_{crystalline}}.100$$ (4)

The 11-point experimental method was employed to determine the biosorbent’s zero-charge point (pHzpc) (Alberti et al., 2023). This approach entails producing 11 solutions with starting pH values ranging from 2 to 12, which are then adjusted with 0.01 M HCl and NaOH. The solutions are then shaken at 30 °C on an orbital shaker for 24 h. Following the contact period, the equilibrium pH values of the materials are determined.

### 2.7 Theoretical models describing adsorption

#### 2.7.1 Kinetic modeling of adsorption processes

Adsorption kinetics research is critical for determining the rate constants and equilibrium periods of adsorption processes essential for efficient pollutant removal. To explore these kinetics, samples of varied dye concentrations were collected at regular intervals under the same conditions as in the equilibrium experiments. The adsorption mechanism is frequently analyzed using kinetic models such as the pseudo-first-order (PFO), pseudo-second-order (PSO), and intraparticle diffusion models (Al-Harby et al., 2021; Belaid and Kacha, 2011). The pseudo-first-order (PFO), pseudo-second-order (PSO), and intraparticle diffusion models were used to examine the adsorption kinetics of MB and CR onto biosorbents made from fish scales. Equations 5–7 respectively, provide the mathematical expressions for these models.

$$q_{t}=q_{e}\;\left({\;1-e}^{t\times K_{1}}\right)$$ (5)

$$q_{t}=\frac{K_{2}\times q_{e}^{2}\times t}{1+K_{2}\times q_{e}\times t}$$ (6)

$${q_{t}=K}_{ID}\times t^{\frac{1}{2}}+C$$ (7)

In adsorption investigations, qe (mg/g) indicates the adsorption capacity at equilibrium, whereas qt (mg/g) indicates the quantity of dye adsorbed at a specific time t. Rate constants K_1_ (1/min) and K_2_ (g/mg·min) are used to characterize the pseudo-first-order and pseudo-second-order kinetic models, respectively. Additionally, C (mg/g) is a constant associated with the thickness of the boundary layer, with larger C values suggesting a greater effect of the boundary layer on the adsorption process, and K_ID_ (mg/g·min^1/2^) is the rate constant for intraparticle diffusion.

#### 2.7.2 Adsorption equilibrium analysis

Adsorption equilibrium data can be described by several models, such as the Langmuir, Freundlich, and Temkin models (Abdelnaeim et al., 2016; Ayawei et al., 2017). The equilibrium equations are provided by the following equation:

$$q_{e}=\frac{q_{m}\times K_{L}\times C_{e}}{1+\left(K_{L}\times C_{e}\right)}$$ (8)

$$q_{e}=K_{F}\times {C_{e}}^{1/n}$$ (9)

$$q_{e}=B_{T}\times \mathrm{Ln}\left(A_{T}\times C_{e}\right)$$ (10)

The variables qe and Ce, respectively, in this equation represent the adsorbate concentrations in the adsorbent and solution. The maximal theoretical adsorption capacity is represented by qm (mg/g). Whereas the Langmuir constant is represented by K_L_(L/mg). The adsorption capability by K_F_(L/g) and the level of the Freundlich isotherm by n. B_T_ = RT/bT, where bT is the Temkin constant linked to the heat of adsorption (J/mol) and A_T_ is the equilibrium binding constant of the Temkin isotherm (L/g).

#### 2.7.3 Adsorption thermodynamics

To evaluate the thermodynamic behavior of MB and CR adsorption on biosorbents, specific equations were used to calculate the Gibbs free energy change (ΔG°). Enthalpy change (ΔH°) and entropy change (ΔS°) (Liyanaarachchi et al., 2023):

$$\mathrm{Ln}\left(\rho K_{c}\right)=\frac{\Delta S{}^{\circ}}{R}-\frac{\Delta H{}^{\circ}}{RT}$$ (11)

$$\Delta G{}^{\circ}=-\text{RT}\times \mathrm{Ln}\left(\rho K_{c}\right)$$ (12)

$$K_{c}=\frac{q_{e}}{C_{e}}$$ (13)

Equations 11 and 12 are used to compute the standard Gibbs free energy change (ΔG°, in J/mol) and the equilibrium thermodynamic parameter (Kc, in L/g). The linear plot of ln(ρKc) versus 1/T was used to measure changes in enthalpy (ΔH°, J/mol) and entropy (ΔS°, J/mol·K), with the slope representing -ΔH°/R and the intercept representing ΔS°/R. R is the universal gas constant (8.314 J/mol·K). ρ is the density of water (g/L), and T is the absolute temperature.

## 3 Results and discussion

### 3.1 Iodine adsorption index (IAI)

A widely used technique for assessing a biosorbent’s microporosity is iodine adsorption (Table 2). Iodine adsorption is improved by raising the temperature; as the temperature climbs from 300 °C to 600 °C, the IAI rises as well. For example, the IAI rises from 301.35 mg/g at 300 °C to 450.16 mg/g at 600 °C with a 1:0.5 impregnation ratio and 1 h of activation. According to this pattern, micropore formation is facilitated by higher temperatures, which improves Iodine adsorption. Similar results, wherein rising temperatures encourage the volatilization of non-carbon species and result in a more developed microporous structure, have been documented in the literature (Chowdhury et al., 2018; Kwiatkowski et al., 2021; Mandzyuk et al., 2013). Longer activation time enhances iodine adsorption, extending the activation period from 1 to 2 h, resulting in an additional rise in iodine adsorption. For example, with a 1:0.5 impregnation ratio and 600 °C, the IAI rises from 450.16 mg/g (1 h) to 510.34 mg/g (2 h); this suggests that a longer activation time leads to improved elimination of volatile materials, resulting in more adsorption sites. This difference can be explained by the influence of the activating agent concentration. At a lower ratio (1:0.5), increasing the activation time promotes the removal of residual volatile compounds and enhances pore development, thus increasing IAI. However, at a higher ratio (1:1), the combined effect of excess phosphoric acid and long activation time can cause over-activation or pore widening, which leads to partial collapse of micropores and a decrease in iodine adsorption capacity. When comparing the results for the 1:0.5 and 1:1 ratio, the 1:1 ratio has a much higher iodine adsorption index. At 600 °C for 1 h. IAI increases from 450.16 mg/g (1:0.5) to 742.28 mg/g (1:1). This shows that a higher concentration of activating agent increases the microporous structure of carbon. The carbon matrix undergoes severe oxidation or gasification at high temperatures and long activation times. This causes the degradation of microporous structures, limiting the available surface area for iodine adsorption. To achieve optimal microporosity, it is essential to regulate the activation time to prevent excessive material loss and the enlargement of pores. To maximize iodine adsorption, a moderate activation duration of around 1 h or a little longer is recommended to avoid compromising the structural integrity.

**TABLE 2:** Summary of preparation parameters and adsorption performance of the biosorbent.

Impregnation ratio	Temperature (°C)	Impregnation time (h)	IAI (mg/g)
1–0.5	300	1	301.35
1–0.5	450	1	346.98
1–0.5	600	1	450.16
1–0.5	300	2	380.76
1–0.5	450	2	470.11
1–0.5	600	2	510.34
1–1	300	1	576.90
1–1	450	1	639.65
1–1	600	1	742.28
1–1	300	2	726.58
1–1	450	2	712.04
1–1	600	2	511.78

The highest IAI (742.38 mg/g) was obtained at 600 °C with 1-h activation and a ratio of 1:1, indicating a well-developed pore structure (Ji et al., 2020). So, we took this SPFS prepared at 600 °C 1:1, and 1 h to conduct a complete study.

### 3.2 Elemental composition of bio-sorbent

The elemental composition of the fish scales and SPFS is similar, as illustrated in Table 3. Carbon (C) levels increased from 18.14% to 23.01%, indicating a rise in organic material in the SPFS post-treatment. This change could be attributed to carbonization, the removal of certain inorganic elements, or the enrichment of organic functional groups (Liu et al., 2024). On the other hand, there was a reduction in Oxygen (O) from 53.36% to 49.30%. This decrease suggests the removal of oxygen-containing compounds, such as hydroxyl groups from hydroxyapatite, potentially due to dehydration or thermal modification (Xin and Shirai, 2021). Phosphorus (P) decreased slightly from 13.94% to 12.15%, indicating a minor loss of phosphate groups, possibly resulting from acid treatment or washing processes. This change may influence adsorption properties since phosphorus is associated with hydroxyapatite (Brahmi et al., 2025). Conversely, calcium (Ca) showed a slight increase from 14.57% to 15.55%, implying that hydroxyapatite is relatively retained within the SPFS, which could enhance metal ion adsorption through the interaction of calcium sites with contaminants (Zhao et al., 2024). The increase in carbon content may enhance adsorption efficiency, while structural modifications suggest improved porosity or increased availability of functional groups for adsorption.

**TABLE 3:** Elemental compositions and molar ratios of FS and biosorbent.

Element (%)	C	O	P	Ca
Fish scales	18.14	53.36	13.94	14.57
SPFS	23.01	49.30	12.15	15.55

### 3.3 FTIR analysis

**FIGURE 2:**
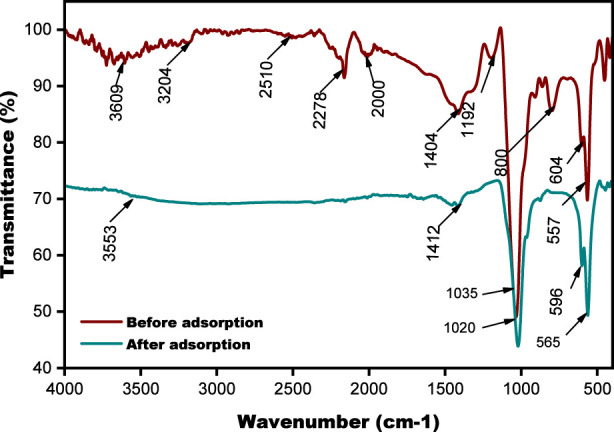
FT-IR spectra of the SPFS before and after adsorption.

The FTIR spectra in Figure 2 present SPFS before adsorption in the wavenumber range 4,000–500 cm^−1^, highlighting the characteristics of various chemical groups through their corresponding bands and peaks. SPFS primarily comprise collagen fibers and hydroxyapatite (Qin et al., 2022). As a result, the surfaces of SPFS are rich in various active functional groups, including hydroxyl, amino, nitro, carbonyl, and phosphate (Yildiz and Yüksel, 2024). The presence of hydroxyl (-OH) groups, most likely from water or collagen in the fish scales, is indicated by the large peak of about 3,200–3,600 cm^−1^, which normally corresponds to O–H stretching vibrations (Marrakchi et al., 2017). Peaks around 2,800–3,000 cm^−1^ indicate C-H stretching in fish scales, possibly due to proteins, lipids, or chemical substances (Ribeiro et al., 2015). The amide peak bands at 1,600–1,700 cm^−1^ and 1,500 cm^−1^ correspond to amide I (C=O stretching) and amide II (N-H bending), which are indicative of collagen, the major protein in fish scales (Kong and Yu, 2007). Peaks at 1,000–1,200 cm^−1^ are commonly associated with phosphate (PO_4_^3-^) groups in hydroxyapatite, a mineral found in fish scales, and C–O groups (Pon-On et al., 2016). Sharp peaks below 1,000 cm^−1^ indicate mineral components such as hydroxyapatite (Ca_10_(PO_4_)_6_(OH)_2_) or carbonate structures (Omoseebi, 2025). These findings are consistent with recently published studies. The FT-IR spectrum of the SPFS changed significantly after adsorption, showing that the active sites and dye molecules successfully interacted (Jaafar et al., 2022). The broad O-H stretching band (3,200–3,500 cm^−1^) shifted and reduced in intensity, showing the involvement of hydroxyl groups in hydrogen bonding with adsorbed dyes (Branca et al., 2016). The peaks at 2,800–3,000 cm^−1^ (C-H stretching) were altered, suggesting interactions with hydrocarbon chains in MB or CR (Saud et al., 2021). The amide I and II bands (1,600–1,700 cm^−1^ and ∼1,500 cm^−1^) showed reduced intensity and minor variation, indicating structural modifications in the collagen matrix, likely due to dye binding (Zhang et al., 2025). Minor shifts in the 1,000–1,200 cm^−1^ range, representing phosphate and C-O groups, may suggest complexation or electrostatic interactions with dye molecules (Arai and Sparks, 2001). Including functional groups enhances SPFS characteristics for MB and CR ions. These findings align with the work of other scholars (Marrakchi et al., 2017).

### 3.4 Scanning electron microscopy (SEM) analysis

**FIGURE 3:**
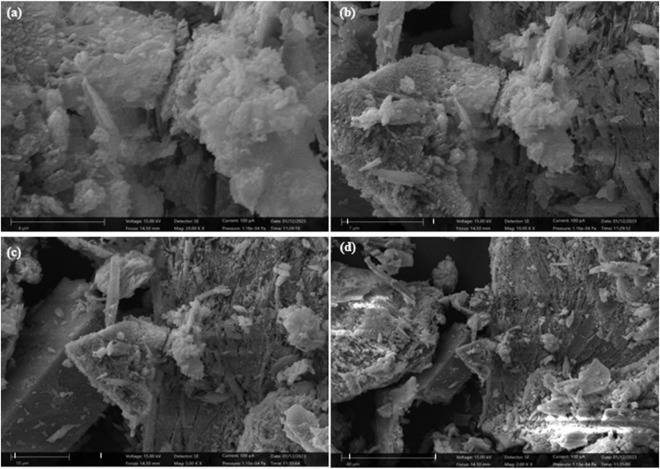
SEM micrographs of SPFS at different magnifications: **(a)** 4 μm, **(b)** 7 μm, **(c)** 10 μm, and **(d)** 40 μm scale bars.

SEM was used to examine and contrast the morphological characteristics of the SPFS, and Figure 3 shows the micrographs that were produced. According to the SEM examination, as shown in images (a) to (d), the SPFS made from fish scales exhibits a complex surface shape with discrete zones, enabling the attachment of MB and CR ions to fiber surfaces (Bortoluz et al., 2020). While the darker sections are rich in proteins, as evidenced by their higher carbon and oxygen content, the lighter areas are rich in minerals like calcium and phosphorus. Its heterogeneous structure enhances the adsorption capacities of the SPFS (Zayadi and Othman, 2013). The surface appears dense and semi-crystalline at close range (Figures 3A,B), with needle-like protuberances scattered throughout globular forms. These microstructures closely resemble the morphologies of phosphate/metal oxide or hydroxyapatite crystals, which are frequently the result of activating H_3_PO_4_ and processing fish scales at high temperatures (Ji et al., 2020). At intermediate magnification (Figure 3C), the surface exhibits noticeable fracture and roughness, along with distinct fissures and the onset of macroporosity. Finally, a highly porous structure with interconnected gaps and laminar pieces is visible at the lowest magnification (Figure 3D), suggesting hierarchical pore networks that can effectively capture big dye molecules (Achieng et al., 2019). The fish scales alter significantly morphologically after being heated. Because of thermo-chemical conversion, charred fish scales seem more compact in SEM pictures than their raw counterparts. The porosity of the material and its adsorption capabilities may be affected by this densification.

### 3.5 Thermal performances

**FIGURE 4:**
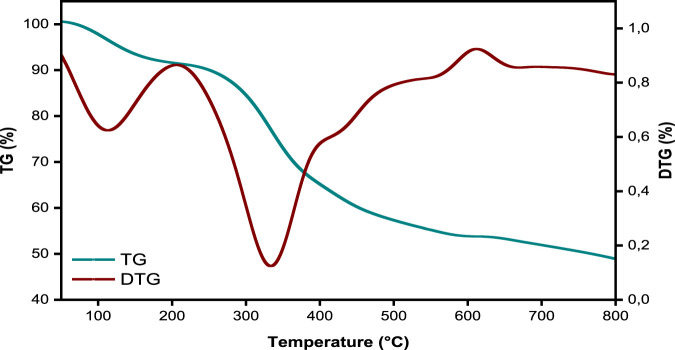
Thermogravimetric analysis (TGA) and derivative thermogravimetry (DTG) profiles of the SPFS.

The TGA and DTA curves in Figure 4 show the SPFS’s thermal response, including dehydration, breakdown, and thermal degradation. Additionally, these curves show important physicochemical alterations, including phase transitions and structural alterations, and they reveal successive stages of mass loss associated with dehydration and decomposition processes (Hadfi et al., 2018). The TG graphs show that the thermal decomposition of fish scales occurred at different temperatures due to differences in the nature and origin of feedstocks (Kasbaji et al., 2023b). At low temperatures (Moisture’s Farewell) (25 °C–210 °C), the mild evaporation of water molecules adhering to the scales’ surface is represented by this phase. An initial mass decrease of about 14.2% is seen when the temperature rises (Babel and Kurniawan, 2003). This weight loss observed between 25 °C and 210 °C corresponds to the progressive removal of physically adsorbed, hydrogen-bonded, and structurally bound water molecules. The upper limit of this range reflects the strong interaction of water within the collagen, hydroxyapatite matrix of the fish scales (Pérez et al., 2025). At temperatures between 200 °C and 500 °C, fish scales lose a significant amount of weight due to the heat breakdown of organic components like collagen and proteins (Milan et al., 2021). Above 500 °C, mass loss becomes slow, indicating breakdown of thermally stable carbonaceous residues and potential partial carbonization (Silva et al., 2019). These findings are corroborated by the DTG curve, which shows a noticeable peak at 332 °C, the maximum rate of thermal degradation. This corresponds to the main stage of degradation that was previously determined by the thermal investigation (Silva et al., 2019). The SPFS can withstand temperatures up to 250 °C, making it appropriate for wastewater treatment at ambient or slightly higher temperatures.

The inclusion of TGA–DTG analysis for the impregnated biosorbent, in addition to the raw material, was essential to assess how chemical modification and inorganic impregnation affect its thermal stability and structural integrity (Morsy et al., 2019). Compared with the raw SPFS, the impregnated sample exhibits a higher residual mass and a slight shift of decomposition peaks toward higher temperatures, demonstrating that the inorganic phase, such as phosphate or metal oxides, enhances the thermal resistance of the carbonaceous matrix (Wang et al., 2025). This stabilization effect confirms the successful incorporation of the inorganic components within the biosorbent structure and their interaction with the organic functional groups (–OH, –COOH, –NH_2_) (Bazarin et al., 2021). Therefore, the TGA–DTG results not only characterize the decomposition stages but also provide evidence of the improved physicochemical robustness and reusability of the modified SPFS for wastewater treatment applications.

### 3.6 X-ray diffraction (XRD) analysis

**FIGURE 5:**
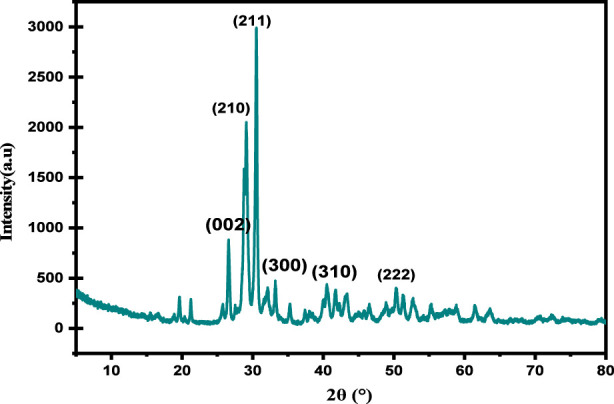
XRD of SPFS.

The prepared SPFS was analyzed using XRD with a 2θ scan from 5° to 90° to evaluate its mineral phases and crystallinity. The XRD curves are shown in Figure 5. The XRD spectrum of the handmade SPFS shows a semi-crystalline structure with a strong and intense diffraction pattern centered near 2θ = 32° and a few peaks at (20° and 40°), corresponding to hydroxyapatite (Ca_10_(PO_4_)_6_(OH)_2_), confirming that the mineral phase remains partially preserved after carbonization, that is the primary mineral ingredient in fish scales, while the broad background hump observed between 2θ = 15° and 35° indicates the presence of amorphous carbonaceous matter and denatured collagen (Azis et al., 2015). The peak of hydroxyapatite indicates that thermal/chemical treatment did not damage or enhance the crystal phases. A lack of peaks in individual bands and a large background hump imply amorphous organic matter, denatured collagen, or damaged protein matrix, which is prevalent in natural biomaterials (Sun et al., 2017). The existence of both crystalline and amorphous phases aligns with the natural composition of fish scales, indicating that the SPFS is semi-crystalline rather than amorphous (Azis et al., 2015). These designs are ideal for adsorption operations since they have a rigid frame for stability and an amorphous region to accept functional groups (e.g., -COOH, -OH, -NH_2_) that bind dye and metal ions.

### 3.7 Identification of the point of zero charge (PZC) of the SPFS

**FIGURE 6:**
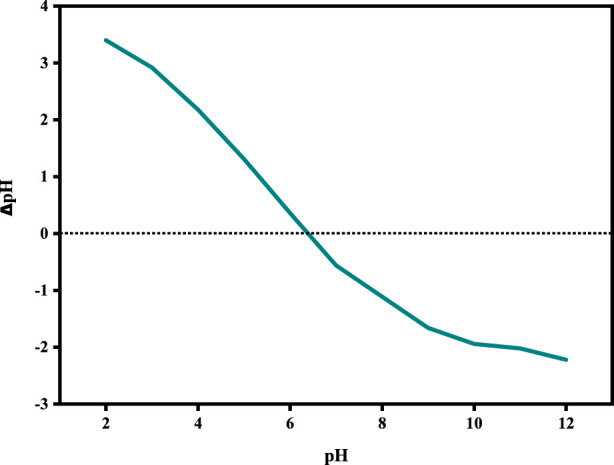
The pH at which the SPFS surface exhibits zero net charge (pH_p_zc).

The point of zero charge (PZC), also known as the isoelectric point (IEP), is the pH point at which the surface of a material has a neutral net charge, indicating that the number of positive and negative surface charges is equal (Parmanbek et al., 2022). The pH_pzc_ of the SPFS was determined to be 6.45, as shown in Figure 6. Indicating that the surface of the SPFS is electrically neutral at this pH. At pH < 6.45, the SPFS’s surface gets charged in the positive direction, making it simpler to adsorb anion species like CR. At pH levels over the pH_PZC_ (pH > 6.45), the surface becomes negatively charged, which promotes electrostatic interaction with cationic contaminants like MB (Kasbaji et al., 2023a). Fish scales are mostly made up of collagen fibers and hydroxyapatite (Ca_10_(PO_4_)_6_(OH)_2_), which may explain their behavior (Salindeho et al., 2022). The active functional groups including amine (-NH_2_) hydroxyl (-OH), and phosphate (-PO_4_^3-^), can be protonated or deprotonated based on the pH of the surrounding solution (Yao et al., 2021). The SPFS effectively removes MB at high pH and CR at low pH, and it has the potential for eco-friendly wastewater treatment applications.

### 3.8 Batch adsorption

#### 3.8.1 Effect of biosorbent dose

**FIGURE 7:**
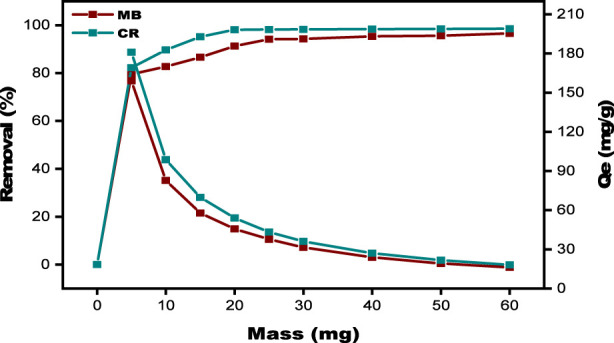
Effect of adsorbent dosage on MB and CR using SPFS.

The effect of biosorbent amount on adsorption efficiency was studied by changing the adsorbent mass from 5 mg to 60 mg. While keeping all other conditions constant, the dye concentration of MB and CR is 20 mg/L, and the contact time is 2 h at the ambient temperature, at an initial pH of 7, and the stirring speed at 300 rpm. Each test used 0.03 g of SPFS. As the total mass of the SPFS increased, the removal efficiency of MB rose from 79.50% to 96.60% and that of CR from 82.26% to 98.56%, as shown in Figure 7. The increase in the adsorbent surface area, which improves interactions with MB and CR molecules, is responsible for this development (Chen et al., 2022). The increased availability of active binding sites on the biosorbent surface, which permits more efficient adsorption interactions between the dye molecules and the SPFS, is primarily responsible for the consistent improvement in dye removal with increased SPFS dosage (Nasrullah et al., 2018). Furthermore, following saturation, the number of sites stabilizes, which could explain why the percentages of removed MB dyes remain stable over time (Kezerle et al., 2018). However, as the SPFS dosage increased, the adsorption capacity (Q_e_) per gram of adsorbent declined, dropping from 159.01 mg/g to 16.10 mg/g for MB and from 164.52 mg/g to 16.42 mg/g for CR. This reduction can be attributed to the diminished solute concentration gradient between the solution and the SPFS surface, which lowers the driving force required for efficient dye uptake (Li et al., 2013). Furthermore, when the mass of the SPFS particles increases, they are more prone to aggregate. Agglomeration reduces the accessible surface area for adsorption, limiting the number of active sites to interact with adsorbate molecules (Deniz and Karabulut, 2017). Furthermore, the observed decrease in adsorption capacity may be caused by additional factors, such as the saturation of adsorption sites and possible changes in surface chemistry during the adsorption process.

#### 3.8.2 Contact time

**FIGURE 8:**
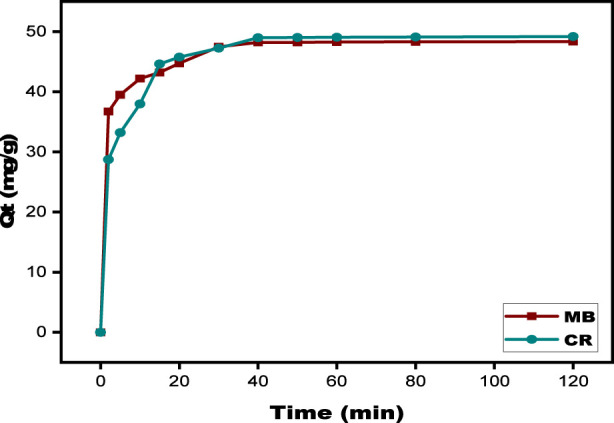
Effect of contact time on MB and CR using SPFS.

The amount of time required for the SPFS to connect with the dye molecules while the adsorption equilibrium is achieved is known as the contact time. The experiments were conducted between 2 and 120 min, while the remaining factors remained constant. As shown in the Figure 8, MB, and CR adsorption onto the SPFS increased dramatically in the first 30 min. This suggests a quick initial adsorption phase, most likely due to external mass transfer, in which dye molecules migrate through the liquid state to the SPFS surface and are held by interfacial contacts and Van der Waals forces (Kasbaji et al., 2023a). The subsequent phase was delayed, indicating internal mass transfer. Rapid surface absorption and slow interparticle diffusion in the SPFS are the main characteristics of SPFS that remove MB and CR. The adsorption of MB and CR reached equilibrium in around 30–40 min, indicating a quick adsorption process. Adsorption capabilities for MB increased from 79.50 mg/g to 96.60 mg/g whereas CR increased from 57.43 mg/g to 98.34 mg/g. Beyond this time, the quantities of MB and CR adsorbed remained steady for 120 min, indicating that the available active sites on the SPFS surface had become saturated and no further adsorption occurred (Wei et al., 2023). A contact time of 60 min appears suitable for achieving equilibrium.

#### 3.8.3 Effect of pH

**FIGURE 9:**
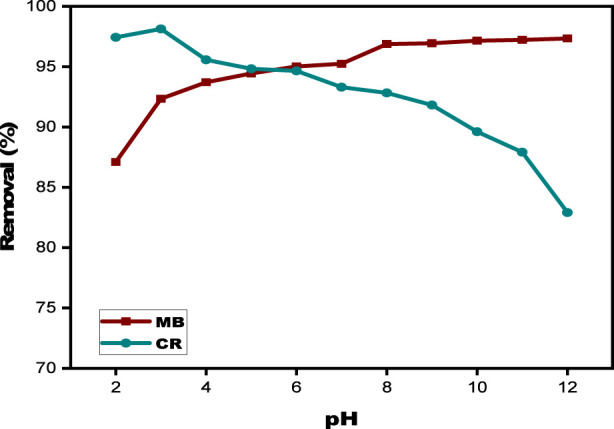
Effect of pH on MB and CR using SPFS.

The pH of the aqueous solution plays a crucial role in dye adsorption by influencing the surface charge of the adsorbent and the ionization behavior of the dye (Sakr et al., 2015). The pH of the solution was varied using 0.1 N HCl and 0.1 N NaOH during the experiments, which were carried out over a pH range of 2–12. All other experimental parameters remained constant. The effect of pH on the biosorption of CR and MB by the SPFS is depicted in Figure 9. The findings show that when the pH rises from 2 to 12, the elimination effectiveness of MB increases dramatically, from 87.09% to 97.34%. On the other hand, throughout the same pH range, the removal effectiveness of CR drops from 97.43% to 82.9%, demonstrating how each dye’s adsorption process is pH-dependent. MB exhibits best removal effectiveness in the neutral to alkaline range (pH 6–12). CR exhibits optimal adsorption in the acidic range (pH 2–3). The reason for this phenomenon can be attributed to pH-dependent variations in surface charge. At low pH levels, the concentration of hydrogen ions is high, giving SPFS surfaces a more positive charge by protonating their active functional groups. In the case of CR, an anionic dye, the positively charged environment encourages electrostatic attraction, which increases adsorption capacity. The similarly charged pearl surface repels MB, a cationic dye, resulting in competition between positively charged patches and MB cations (Kerouad et al., 2025). As pH rises, the positive surface charge of the SPFS diminishes, reducing affinity for CR while increasing MB adsorption, resulting in lesser CR elimination but higher MB absorption (Ahmad and Ansari, 2021; Gupta et al., 2020). The reason for this phenomenon is that at higher pH values, the SPFS surface has more negatively charged functional groups, which increases electrostatic attraction to the positively charged dye cations and increases their adsorption efficiency. These results show that when the pH increases higher than the point of zero charge, the SPFS surface gains a negative charge, which is in line with the patterns seen regarding pH. Because of the greater electrostatic interactions, this state promotes the uptake of cationic dyes like MB. To guarantee the best adsorption effectiveness, further tests were carried out at pH 3 for CR and pH 6 for MB in light of these findings. When both Methylene Blue (MB) and Congo Red (CR) coexist in the same aqueous solution, the adsorption behavior of each dye becomes competitive and strongly dependent on the surface charge of the SPFS. Based on individual dye studies, CR favors acidic conditions (pH 2–3), while MB performs best at neutral to slightly alkaline conditions (pH 6–12). In a mixed system, an intermediate pH of approximately 5 ± 1 offers a compromise, where the biosorbent surface possesses both positively and negatively charged sites. This dual charge character facilitates the electrostatic attraction of both cationic and anionic dyes. Additionally, π–π interactions and hydrogen bonding further support the simultaneous uptake. Therefore, maintaining the pH near (6–7) represents the most effective condition for the combined removal of both dyes in practical wastewater treatment applications.

#### 3.8.4 Influence of initial dye concentration

**FIGURE 10:**
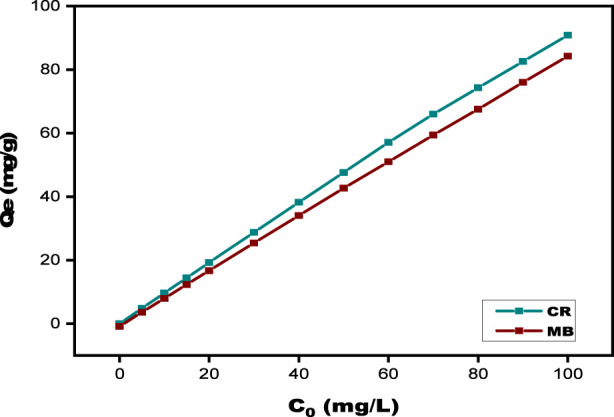
Effect of initial dye concentration on MB and CR using SPFS.

To investigate how the initial concentrations of MB and CR influence adsorption efficiency, all other parameters were maintained constant throughout the tests, which used dye doses that varied from 5 to 100 mg/L. The correlation between the starting dye concentration and the corresponding removal efficiency for both MB and CR is shown in Figure 10. The kinetic graphs demonstrate that as the initial concentrations of MB and CR are increased, the matching qe increases in tandem with the MB and CR rates. For MB, it rose from 4.86 to 90.81 mg/g while for CR, it rose from 4.86 to 92.80 mg/g. The initial concentrations of MB and CR act as the driving force that promotes the mass transfer of dye molecules by overcoming the resistance between the liquid phase and the surface of the solid SPFS (Ahmad and Ansari, 2021; Sharma et al., 2022). The concentration gradient rises with increasing initial MB and CR concentrations, enhancing the adsorption driving force. Because there are more collisions at higher concentrations, there is a greater chance that the dye molecules and the SPFS surface may interact. Importantly, the initial concentration of MB and CR dyes plays a key role in minimizing mass transfer limitations between the liquid phase and the target pollutants during the adsorption process (Gupta et al., 2020). In addition, larger initial MB and CR concentrations resulted in increased starting taking in efficiency. All dye ions may easily reach the active functional groups on the SPFS surface at reduced dye concentrations, but because there are fewer molecules available, removal efficiencies may be comparatively poor. The availability of dye ions, on the other hand, increases retention efficiency at greater concentrations, but only to a certain extent. After that, the adsorption sites become saturated, and the removal efficiency approaches a plateau (Alver et al., 2020).

#### 3.8.5 Effect of temperature

**FIGURE 11:**
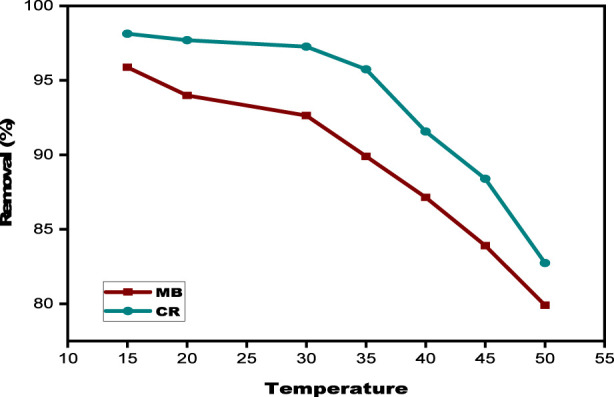
The effect of temperature on MB and CR using SPFS.

To determine the optimal temperature for the bioadsorption process, experiments were conducted at various temperatures (15, 20, 30, 35, 40, 45, and 50 °C) to evaluate their influence on the adsorption capacity of the SPFS for MB and CR. A mass of 0.03 g of SPFS, 20 mL of dyes, and 120 min was used in the studies. The percentage of MB and CR eliminated for temperature is displayed in Figure 11. However, both MB and CR lose dye retention while the temperature increases from 15 °C to 50 °C. Given that higher adsorption effectiveness is favored by lower temperatures, this tendency implies that the bioadsorption procedure is exothermic. The decreasing strength of physical connections between dye molecules and functional groups on the SPFS surface, such as hydrogen bonds and van der Waals forces, could explain the performance loss at higher temperatures. These outcomes are consistent with the conclusions of the published research (Mukkanti and Tembhurkar, 2021; Nakro et al., 2025). Moreover, both dyes became more soluble, indicating stronger interactions between the solvent and solute. As the interactions between bio-adsorbents and colorants decrease, dye ions in the medium are less likely to be adsorbed by the biosorbents (Gupta et al., 2020). As the temperature increases from 15 °C to 50 °C, the clearance rate for MB and CR decreases from 95.88% to 79.90% and from 98.13% to 82.73%, respectively. This enables us to deduce that ambient temperature produces the best results.

#### 3.8.6 Kinetic studies

**FIGURE 12:**
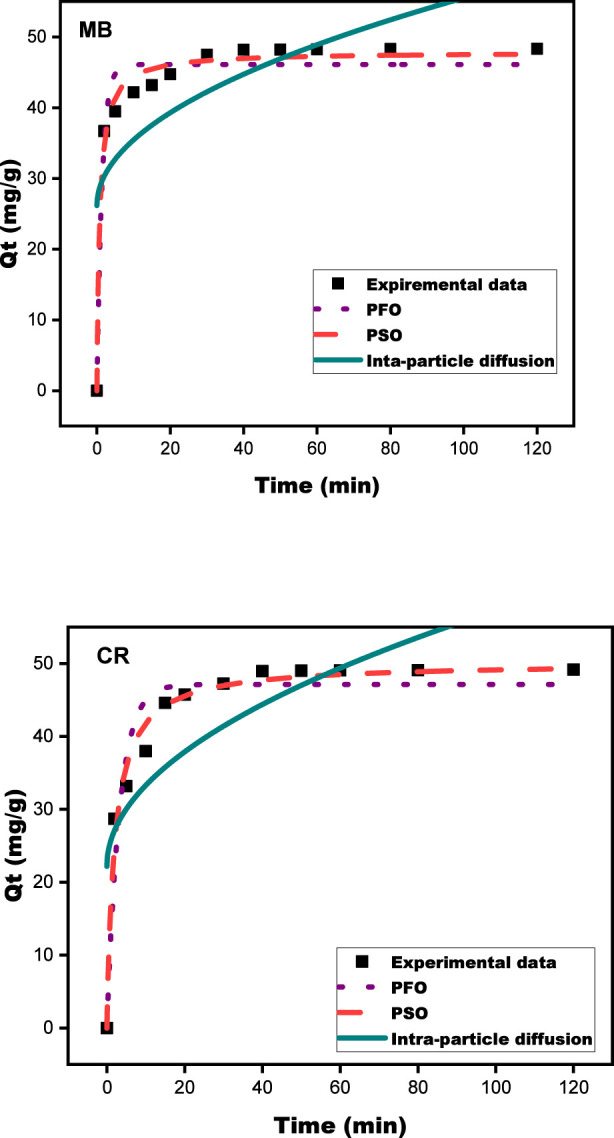
Pseudo-first-order, pseudo-second-order, and intraparticle diffusion kinetic models applied to the adsorption of MB and CR onto the SPFS.

Kinetic parameters are critical for designing and modeling the adsorption process and comprehending adsorption dynamics concerning the order of the rate constant (Nakro et al., 2025). Models such as PFO, PSO, and intraparticle diffusion were employed to evaluate the experimental data for MB and CR adsorption on SPFS, as shown in Table 4. The assessment of the adsorption kinetics is shown in Figure 12. The strong correlation coefficients (*R* ^2^ = 0.985 for MB and *R* ^2^ = 0.982 for CR) show that, of the examined models, the pseudo-second-order (PSO) model best fits the experimental data for both MB and CR. It involves the many stages of the absorptive route, including the adsorption itself, intraparticular diffusion, and external diffusion. Additionally, the qt data collected for MB and CR are consistent with the experimental qt results. This implies that chemical interactions, such as the transfer of electrons or binding by covalent bonds through electron exchange throughout the dye molecules and the SPFS surface, are probably what control the total adsorption rate for MB and CR (Saini et al., 2021). Therefore, without implying the identification of the exact involved mechanisms, the application of this template suggests that the adsorption operation can be driven by a chemical method. Furthermore, intraparticle diffusion plays a significant role in the removal of MB and CR from the SPFS, especially after the initial 60 min. This transitory phase is caused by the dye molecules gradually passing through the complicated porous network of activated carbon. Once the dyes have diffused into the micropores and mesopores and reached the interior active sites, they are gradually adsorbed and stacked along the internal pore surfaces. Even though the chemical reaction on the surface begins within the first few minutes (following a PSO) chemical model with excellent adjustment and a high *R* ^2^ value, the most important factor in the long run is still intra-particular diffusion because it regulates and controls the rate at which the colorants are captured at each moment (Kasbaji et al., 2024). Notably, other scientists have gotten similar template settings for a variety of other adsorptive materials (Ahmad and Ansari, 2021; Sharma et al., 2022).

**TABLE 4:** Kinetic modeling of MB and CR adsorption onto SPFS using different approaches.

Model	Parameters	Type of dye	
		MB	CR
PFO	q_t.exp_ (mg/g)	48.326	49.173
	q_t.cal_ (mg/g)	46.103	47.145
	K_1_ (min^−1^)	0.714	0.306
	*R* ^2^	0.955	0.933
PSO	q_t.cal_ (mg/g)	47.870	50.111
	K_2_ (g/mg * min)	0.026	0.010
	*R* ^2^	0.985	0.982
Intra-particle diffusion	K_ID_ (mg/g min^−1/2^)	2.933	3.510
	C	26.171	22.191
	*R* ^2^	0.488	0.579

#### 3.8.7 Equilibrium studies

**FIGURE 13:**
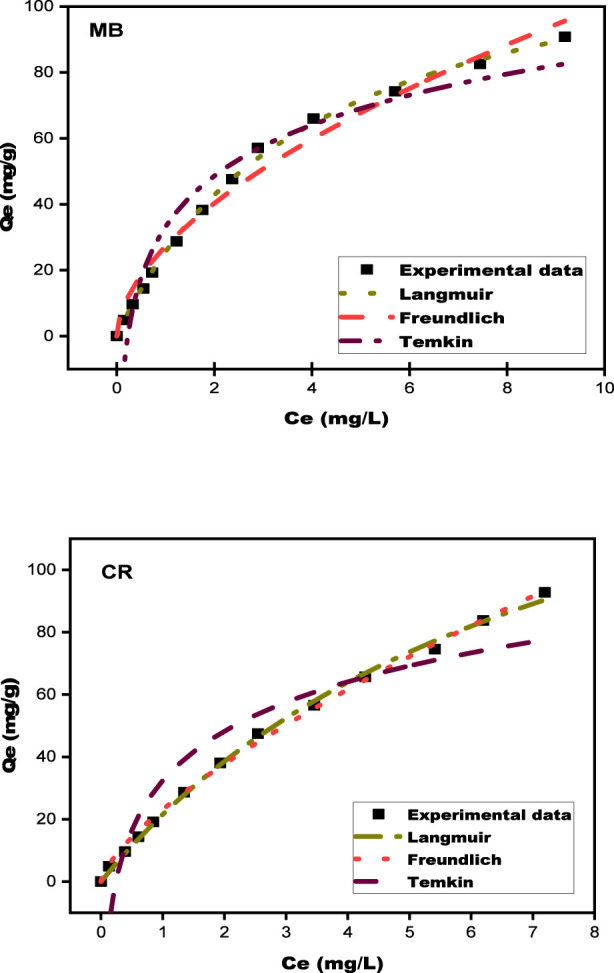
Langmuir, Freundlich, and Temkin isotherm plots for MB and CR adsorption onto SPFS.

The adsorption behavior of MB and CR on biosorbents was investigated using the Langmuir, Freundlich, and Temkin isotherm models, as shown in Figure 13. Table 5 outlines the corresponding parameters. These models help establish the best match for the experimental data and identify the optimum adsorption capabilities. Figure 13 depicts the equilibrium distribution patterns for the two dyes on the SPFS. The study used three isotherm models to describe dye removal behavior. The results showed a significant increase in adsorption at lower dye concentrations, indicating a strong connection between the dyes and the SPFS. The Langmuir isotherm showed the strongest association with experimental data, with *R* ^2^ values approaching 1. This tight match implies that adsorption happens uniformly across the biosorbent surface, with monolayer coverage and isoenergetic binding sites, and with little interaction between adsorbed molecules (Ragadhita and Nandiyanto, 2021). At higher concentrations of the dyes MB and CR, multilayer adsorption may occur, as evidenced by the low correlation with the Freundlich isotherm model. The Freundlich constant *n*F indicates the intensity and strength of adsorption interactions, with values of 1.765 for MB and 1.412 for CR (Guerrero-fajardo et al., 2025). Given that both values are greater than 1, it is confirmed that both dyes benefit from the adsorption process. Moreover, the adsorption is physically favorable, in accordance with a physisorption process, as 1/n_F_ <1 in both cases (about 0.566 for MB and 0.708 for CR). The adsorption of MB and CR onto the biosorbent is advantageous, as evidenced by the separation factor R_L_ values, which are computed using the Langmuir model and lie between 0 and 1. Particularly at higher dye concentrations, this implies an initial chemisorption interaction that may be followed by physisorption (Suguna et al., 2010). The Temkin equilibrium, which takes into consideration the influence of adsorbent-adsorbate interactions, produced a good fit for the adsorption of MB and CR onto the SPFS. The correlation coefficients (*R* ^2^) for MB and CR were 0.953 and 0.907, respectively, indicating moderate agreement, although lower than those obtained with the Langmuir and the Freundlich models. The Temkin model represents the exothermic character of the adsorption process, as demonstrated by the positive Temkin constants (BT) of 22.295 for MB and 22.923 for CR, which relate to the heat of adsorption. Table 6 summarizes the comparison of SPFS Qmax to various biosorbents described in prior work on MB and CR. Table 6 shows that biosorbent is a promising material for removing MB and CR from aqueous media. It has a higher adsorption capacity than other biosorbents. Despite its importance in capturing the energetic components of dye uptake, the Temkin model appears to be less appropriate for characterizing the entire adsorption behavior than the Freundlich model, which better accounts for surface heterogeneity and the multilayer structure of adsorption.

**TABLE 5:** Langmuir, Freundlich, and Temkin methods for removing MB and CR from SPFS.

Model	Parameters	Type of dye	
		MB	CR
Langmuir	q_max_ (mg/g)	129.694	187.634
	K_LF_ (min^−1^)	0.246	0.129
	R_L_	0.169	0.279
	*R* ^2^	0.998	0.998
Freundlich	K_F_ (mg/g) (L/mg)1/n	27.229	23.089
	n_F_	1.765	1.412
	*R* ^2^	0.981	0.997
Temkin	B_T_	22.295	22.923
	A_T_	4.424	4.099
	*R* ^2^	0.953	0.907

**TABLE 6:** Comparing the experimental adsorption performance of MB and CR using different types of adsorbents.

Adsorbent	Adsorbate	Adsorption conditions (pH, C, T)	Adsorption capacity Qm (mg/g)	References
Fish-scale-based porous carbon	MB	pH = 7.07; C_0_ = 200 mg/L; T = 30 °C	555.55	Huang et al. (2014)
Activated carbon derived from fish scales	CR	pH = 4; C_0_ = 20 mg/L; T = 25 °C	19.58	Nakro et al. (2025)
Biosorbent from fish scales	MB	C_0_ = 100 mg/L; T = 25 °C	74.96	Ghazoui et al. (2024)
SPFS	CR	pH = 3; C_0_ = 20 mg/L; T = 30 °C	187.634	This study
SPFS	MB	pH = 6; C_0_ = 20 mg/L; T = 30 °C	129.694	This study

#### 3.8.8 Thermodynamic parameters

**FIGURE 14:**
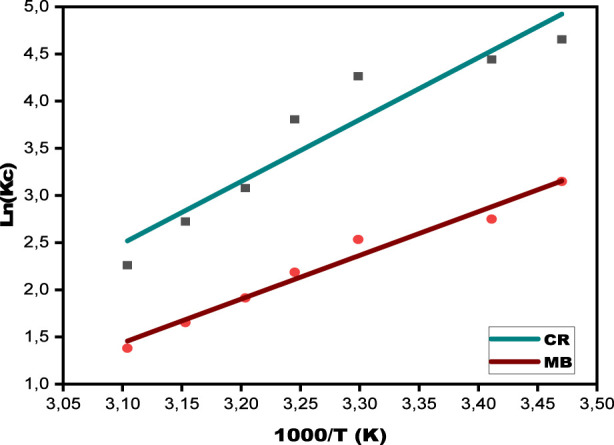
Plots of ln(Kc) against 1/T for the elimination of the MB and CR by SPFS.

A crucial component of sorption investigations is evaluating thermodynamic characteristics, which aid in identifying whether physical or chemical interactions control the adsorption process (Laghzal et al., 2024). The thermodynamic behavior of CR and MB adsorption was examined in this work throughout a temperature range that varied from 288.15 K to 322.15 K. A plot of ln (Kc) vs. 1,000/T was created to extract the pertinent thermodynamic parameters, as shown in Figure 14, which provides information about the nature and spontaneity of dye removal utilizing the SPFS. The graph’s slope was used to determine the enthalpy change (ΔH◦), and the intercept was used to get the entropy change (ΔS◦). After determining the enthalpy and entropy values. Equation 12 was used to calculate the Gibbs free energy values (ΔG◦) for each of the five temperatures (Kasbaji et al., 2023a). Table 7 displays all of the findings. Both colors have negative enthalpy values, indicating that the dye removal process on the SPFS is exothermic. As a result, the enthalpy values obtained from thermodynamic analysis can be utilized to determine the sort of interaction, physical or chemical, between the SPFS functional groups and the dye molecules. In general, an enthalpy shift below 40 kJ/mol suggests that physical forces, not chemical bonds, are responsible for the adsorption process (Kasbaji et al., 2023b). On the other hand, a value exceeding 40 kJ/mol indicates chemical sorption. Thus, figuring out the enthalpy value is crucial to figuring out what kind of adsorption is taking place (Lima et al., 2019). An enthalpy below 40 kJ/mol for SPFS indicates physical adsorption, dominated by low Van der Waals forces. The negative values of ΔG° for MB and CR, which decrease with temperature, confirm a spontaneous and favorable process. The adsorbent surface interactions are more organized when the ΔS° is negative. These thermodynamic findings demonstrate that the SPFS’s spontaneous, exothermic, and physically regulated removal of MB and CR occurs.

**TABLE 7:** Thermodynamic parameters involved in the removal of MB and CR from the examined SPFS.

Type of dye	ΔG° (KJ mol^-1^)							ΔH° (KJ mol^−1^)	ΔS° (kJ.mol^−1^ K^−1^)
	288.15 K	293.15 K	303.15 K	308.15 K	312.15 K	317.15 K	322.15 K		
MB	−7.53	−6.70	−6.38	−5.59	−4.96	−4.35	−3.69	−38.52	−107.47
CR	−11.14	−10.82	−10.74	−9.74	−7.98	−7.18	−6.05	−54.56	−148.44

#### 3.8.9 Recyclability of the biosorbents

**FIGURE 15:**
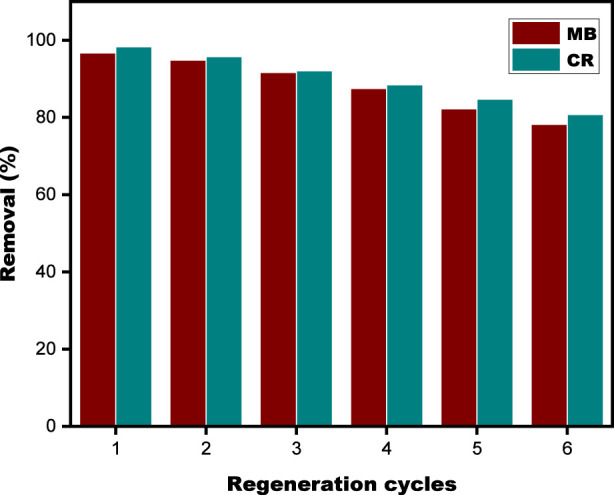
The SPFS’s histogram of MB and CR removal rates throughout various adsorption cycles.

The SPFS was subjected to 50 mL of a 1 M HCl solution for 30 min at ambient temperature during the desorption phase. Figure 15 displays the results of the adsorption and desorption cycles. According to the histogram, the eliminated area of MB and CR somewhat declines as the number of reusability test runs increases. This decrease in adsorption capacity is likely due to the gradual accumulation of dye residues on the SPFS surface, which are not completely removed during desorption, resulting in reduced efficiency after several reuse cycles (Gupta et al., 2020). The SPFS’s excellent adsorption performance for both pollutants was sustained for at least six consecutive cycles without a noticeable decrease in efficiency because of its efficient regeneration capability. In summary, the SPFS demonstrated themselves to be dependable, effective, and eco-friendly substitutes for the elimination of CR and MB. It is also important to note that using these materials for bioremediation can aid in lowering waste from fish scale processing, a problem that presents increasing environmental, societal, and economic difficulties in many regions of the world.

## 4 Conclusion

An ecological SPFS has been developed from activated carbon derived from fish scales, thus enhancing an abundant natural resource often considered waste. This material has a porous surface rich in active sites, offering remarkable adsorption capacity for MB and CR dyes, with around 400 mg/g capacities. The richness in functional groups such as carbonyls, hydroxyl, and carboxylic groups derived from the protein and mineral matrix of scales facilitates interaction with colouring molecules, thus optimizing removal efficiency. The adsorption performance remains high (above 95%) over a wide range of concentrations and temperatures, demonstrating a remarkable robustness of the bioabsorber. In addition, the material retains its efficiency after several cycles of regeneration, highlighting its durability and potential for environmentally friendly industrial use. The Langmuir-Freundlich adsorption model is best suited to experimental data, reflecting single-layer adsorption on a heterogeneous surface. Kinetics reveal a chemisorption involving complex ionic interactions. Thermodynamic analysis confirms that adsorption is a spontaneous, exothermic, and physically favorable process. This study illustrates the promising path opened by fish scale biosorbent, offering an ecological, efficient, and innovative solution for wastewater remediation and sustainable exploitation of natural resources.

## Data Availability

The raw data supporting the conclusions of this article will be made available by the authors, without undue reservation.
